# Supplementary material for: Acute pancreatitis in children – morbidity and outcomes at 1 year
Source: BMJ Paediatr Open. 2022 Jul 5;6(1):e001487. doi: 10.1136/bmjpo-2022-001487 (PMC9258515; doi:10.1136/bmjpo-2022-001487)
Supplement: Author's manuscript [file bmjpo-2022-001487.draft_revisions.pdf]

BMJ Paediatrics Open

BMJ Paediatrics Open is committed to open peer review. As part of this commitment we make the peer review history of every article we publish publicly available.

When an article is published we post the peer reviewers' comments and the authors' responses online. We also post the versions of the paper that were used during peer review. These are the versions that the peer review comments apply to.

The versions of the paper that follow are the versions that were submitted during the peer review process. They are not the versions of record or the final published versions. They should not be cited or distributed as the published version of this manuscript.

BMJ Paediatrics Open is an open access journal and the full, final, typeset and author-corrected version of record of the manuscript is available on our site with no access controls, subscription charges or pay-per-view fees (http://bmjpaedsopen.bmj.com).

If you have any questions on BMJ Paediatrics Open's open peer review process please email info.bmjpo@bmj.com

BMJ Paediatrics Open

Long term morbidity and outcomes of acute pancreatitis in children

Journal:	*BMJ Paediatrics Open*
Manuscript ID	bmjpo-2022-001487
Article Type:	Original research
Date Submitted by the Author:	06-Apr-2022
Complete List of Authors:	Bhanot, A; University of Bristol, Faculty of Health Sciences,<br>Majbar, AA; Sabratha Teaching Hospital, Department of Paediatrics<br>Candler, Toby; University Hospitals Bristol NHS Foundation Trust, Paediatric Diabetes and Endocrinology<br>Hunt, LP; University of Bristol, NIHR Bristol Biomedical Research Centre, Nutrition Theme<br>Cusick, E; University Hospitals Bristol NHS Foundation Trust<br>Johnson, Paul; University of Oxford, Nuffield Department of Surgery; NIHR Oxford Biomedical Research Centre<br>Shield, Julian; University of Bristol, Biomedical Research Unit in Nutrition; University Hospitals Bristol NHS Foundation Trust, Paediatric Diabetes and Endocrinology
Keywords:	Endocrinology, Gastroenterology

SCHOLARONE™
Manuscripts

I, the Submitting Author has the right to grant and does grant on behalf of all authors of the Work (as defined in the below author licence), an exclusive licence and/or a non-exclusive licence for contributions from authors who are: i) UK Crown employees; ii) where BMJ has agreed a CC-BY licence shall apply, and/or iii) in accordance with the terms applicable for US Federal Government officers or employees acting as part of their official duties; on a worldwide, perpetual, irrevocable, royalty-free basis to BMJ Publishing Group Ltd ("BMJ") its licensees and where the relevant Journal is co-owned by BMJ to the co-owners of the Journal, to publish the Work in this journal and any other BMJ products and to exploit all rights, as set out in our licence.

The Submitting Author accepts and understands that any supply made under these terms is made by BMJ to the Submitting Author unless you are acting as an employee on behalf of your employer or a postgraduate student of an affiliated institution which is paying any applicable article publishing charge ("APC") for Open Access articles. Where the Submitting Author wishes to make the Work available on an Open Access basis (and intends to pay the relevant APC), the terms of reuse of such Open Access shall be governed by a Creative Commons licence – details of these licences and which Creative Commons licence will apply to this Work are set out in our licence referred to above.

Other than as permitted in any relevant BMJ Author's Self Archiving Policies, I confirm this Work has not been accepted for publication elsewhere, is not being considered for publication elsewhere and does not duplicate material already published. I confirm all authors consent to publication of this Work and authorise the granting of this licence.

Confidential: For Review Only

One year follow up of children diagnosed with acute pancreatitis: co-morbidity and outcomes.

Journal: Archives of Disease in Childhood

Title (max. 10 words)**:** Long term morbidity and outcomes of acute pancreatitis in children

Authors: Bhanot A[*1], Majbar AA[*2], Candler TP[3], Hunt LP[4], Johnson PRV[56], Cusick E[3], Hamilton-Shield J[3,4].
*** Joint first Authors**
Word count: 2691 words
Abstract: 240 words
Tables/illustrations: 5
References: 37

1. Faculty of Health Sciences, University of Bristol, BS2 8DZ, UK
2. Department of Paediatrics, Sabratha Teaching Hospital, Sabratha, Libya
3. Bristol Royal Hospital for Children, Upper Maudlin Street, Bristol, BS28BJ
4. NIHR Bristol Biomedical Research Centre, Nutrition Theme, University of Bristol, BS1 2NT, UK
5. Academic Paediatric Surgery Unit, Nuffield Department of Surgical Sciences, University of Oxford, John Radcliffe Hospital, Oxford
6. NIHR Oxford Biomedical Research Centre

Correspondence to: Professor Julian Hamilton-Shield, Bristol Royal Hospital for Children, Upper Maudlin Street, Bristol, BS2 8BJ. or e-mail: j.p.h.shield@bristol.ac.uk

Confidential: For Review Only

Abstract

Objective:

To establish short- and medium-term complications one year post diagnosis, of acute pancreatitis (AP) in children aged 0 to 14 years.

Design:

One year follow-up of a prospective monthly surveillance of new cases of AP in children under 15 years through the British Paediatric Surveillance Unit (BPSU) from April 2013 to April 2014.

Setting:

A monthly surveillance of >3700 consultant paediatricians and paediatric surgeons in the United Kingdom and Ireland using the BPSU.

Patients:

Children aged 0-14 years with a new diagnosis of AP.

Main outcome measures:

The outcomes following AP, including the incidence of complications and co-morbidity at diagnosis and at one year.

Results:

Of the 94 new confirmed cases of AP identified in the UK during the study period, 90 cases (96%) were included in the one-year follow-up. 30 patients (32%) developed further episode(s) of AP. Over a fifth of patients developed one or more major complication. At initial admission, the most common of these was pancreatic necrosis (n=8, 9%), followed by respiratory failure (n=7, 7%). Reported complications by one year were pseudocyst formation (n=9, 10%), diabetes requiring insulin therapy (n=4, 4%) and maldigestion (n=1,

Confidential: For Review Only

1%). At one year post diagnosis, only 59% of children had made a full recovery without residual problems. Two patients died, indicating a case fatality of ~2.0%.

Conclusions:

Acute pancreatitis in childhood is associated with significant short- and medium-term complications and co-morbidities including risk of recurrence in approximately a third of cases.

Confidential: For Review Only

Introduction

Acute pancreatitis (AP) is an inflammatory condition classically presenting with sudden onset upper abdominal pain.[1] The first prospective study of AP in UK children under 15 years found an incidence of 0.78 per 100,000.[2] It is reported that 25% of children develop major complications following an episode of AP.[3,4] The clinical course and complications of AP in children are variable, ranging from mild self-limiting illness to organ failure and death. [5,6]

Pancreatic necrosis, pseudocyst formation and abscesses are some of the complications following AP.[6] Diabetes Mellitus (DM) after AP has also been reported in children, with one retrospective study in the United States reporting DM in 4.5% of cases.[7] Until now in the UK, there has been no prospective study focusing on the complications and co-morbidity following AP in children.

Following an episode of AP, children may develop further episodes.[5] Recurrence has been reported in 15–35% of children with AP.[6] One prospective study in the United States found that 17% of patients developed acute recurrent pancreatitis following an episode of AP.[8] However, the rate of recurrence following AP has yet to be determined in a prospective UK study.

The aim of this study was to conduct the first prospective follow-up to a national-based analysis of AP in childhood to characterise the outcomes of AP, including determining the prevalence of complications and co-morbidity in the short to medium term.

Confidential: For Review Only

Methods

As previously described, a prospective study was carried out using the British Paediatric Surveillance Unit (BPSU) reporting system of the Royal College of Paediatrics and Child Health (RCPCH) to identify any new diagnosis of AP in 0-14 year old children in the UK and Ireland.[2] From April 2013 to April 2014, AP in children under the age of 15 years was included on the orange card sent monthly to all consultant paediatricians who undertake clinical work in the UK or the Republic of Ireland. To identify children directly referred to surgical departments, the British Association of Paediatric Surgeons (BAPS) supported this study and paediatric surgeons received the orange card during the study period.

Diagnosis of AP required at least two of the three following features: (1) Acute onset of upper abdominal pain; (2) Serum amylase and/or lipase raised ≥3 times the upper limit of normal local range; and (3) Imaging findings characteristic of AP. These criteria for the diagnosis of AP were adapted according to revised Atlanta criteria by the "Acute Pancreatitis Classification Working Group" and a consensus of the International Study Group of Paediatric Pancreatitis: In Search for a Cure (INSPPIRE)[9,10]. Clinicians returned the card notifying the BPSU of any AP cases seen or "nothing to report". The orange card return compliance was 95.3% during the study.

Each reporting clinician was sent an initial questionnaire to collect additional information on the clinical presentation, diagnosis, and management, as well as acute complications and mortality. Upon the questionnaire's completion, the eligibility of the patient was determined and the physicians' diagnoses were reviewed by the study investigators (A.A.M, J.P.H.S, E.C).[2] If a child had more than one episode of AP during the study period, only the

Confidential: For Review Only

first episode was included as a new case and the information on following episode(s) was used as follow-up data.

A second questionnaire was sent to the reporting clinician one year after the initial presentation for diagnostic review, establish management patterns, and identify associated morbidity and outcome. The follow-up questionnaire had four sections. Section A collected information about weight and height. Section B collected data on medium term complications. Section B1 asked about any new surgical interventions, as well as development of pseudocyst, its diagnosis and treatment. Section B2 asked about the development of diabetes, and/or maldigestion and its diagnosis and treatment. Section C detailed further investigations undertaken after initial investigation and Section D asked about recurrence and outcome. The initial questionnaire also collected data on acute complications and mortality.

Statistics

Data were analysed using the Statistical Package for the Social Sciences (SPSS) (Version 21.0. Armonk, NY: IBM Corp), Stata vs 12.1 (StataCorp LP, Texas, 1985-2011) and Microsoft Excel (2013).

The proportions of patients who were considered to have mild or moderate/severe disease were determined. Factors associated with developing AP were compared between mild and moderate/severe groups using Fisher's Exact test.

The percentages of patients developing short and medium-term complications up to one year post diagnosis of the first episode of AP were determined. Criteria for considering

https://mc.manuscriptcentral.com/bmjpo

Confidential: For Review Only

major complications of the first episode were: (1) all complications required to classify an episode as moderate/severe pancreatitis (organ failure, pancreatic necrosis, and pseudocyst formation), (2) complications requiring treatment at the initial admission (hyperglycaemia) and (3) residual disorders requiring treatment at the end of the follow-up period (diabetes and maldigestion). The major complications were divided into short-term (identified early during initial admission) and medium term (identified in patients with prolonged admission or after discharge during follow-up). Minor complications were also reported.

The percentage of patients who developed further episode(s) of AP within one year from diagnosis were identified, as well as the average number of recurrent episodes and reported associations.

The prognosis and medium-term outcomes at one year of the first episode of AP in children were evaluated by dividing the cohort into five outcome groups: (1) children with full recovery, (2) children with full recovery from the initial episode, but with recurrence of pancreatitis, (3) children with residual problems without recurrence, (4) children with residual problems and recurrence, and (5) children who died while hospitalised.

Ethics

The study was approved by the National Research Ethics Service Committee South West, Central Bristol (REC reference 11/SW/0132) and was granted Section 251 of the National Information Governance Board for Health and Social Care permission by its Ethics and Confidentiality Committee under reference ECC 6-02(FT12)/2012.

Confidential: For Review Only

Results

From April 2013 to April 2014, there were 94 new confirmed cases of AP identified. At one year follow-up, clinical data were available for 90 of these cases (96%), no responses were received for four cases.

Demographics

Female to male ratio was equal with 48/94 (51%) male. The median age at diagnosis was 11.2 years (IQR 7.1-14.4), range 1.3 to 14.9 years). White children accounted for 57 of 94 cases (61%), compared to 26 (28%) from Asian and five (5%) from African ethnic groups. Children of Pakistani origin accounted for the majority (18 of 26; 69%) of the patients of Asian heritage and 19% of the total cohort.[2]

Outcomes

Sixteen (17%) of the 94 new cases were considered to have moderate/severe disease. Table 1 shows the main associations with mild and moderate/severe cases showing no significant differences in the main associations between the two severity groups (P=0.22). Two children (2.1%) died in hospital during their first admission: both had underlying chronic conditions (propionic acidaemia and cerebral palsy, global developmental delay, epilepsy and microcephaly). All children required hospital management; eight requiring intensive care. Eighty-six patients (91%) were treated conservatively during their initial hospitalisation. Twenty (21%) required one or more related surgical intervention during the initial admission and/or the follow-up period.

https://mc.manuscriptcentral.com/bmjpo

Confidential: For Review Only

The outcomes of the 90 cases included at one year are shown in Table 2. Residual co-morbidities included insulin-dependent diabetes (n=4), clinical and imaging features of chronic pancreatitis (n=3), maldigestion with failure to thrive (n=1) and residual radiological changes in the pancreas without significant clinical findings (n=1).

Recurrent disease

Thirty patients (33%) developed further episode(s) of AP within one year. These 30 had 88 episodes during the study period with an average of 2.9 episodes/patient (Table 3). Twenty-five of these (83%) had mild initial episodes.

The reported associations of AP in the recurrent group were idiopathic (n=13, 43%), genetic (n=5, 17%), drugs (n=5, 17%), organic acidaemias (n=4, 13%), pancreas divisum (n=1, 3%), gallstones (n=1, 3%) and α1-antitrypsin deficiency (n=1, 3%). Each of the five drug-associated cases (two asparaginase, one mercaptopurine, one carbamazepine and one opiates) developed one further episode. The recurrent episode in the child whose first episode was due to opiates was again attributed to opiates. The new episode in the child whose first episode was mercaptopurine-associated was ascribed to prednisolone treatment for acute lymphoblastic leukaemia. For the other three cases, available data did not confirm or discount whether the new episodes developed by reintroducing the same medication.

At least one imaging examination was performed during the first episode in the majority of this recurrent group (28 of 30; 93%). Further investigations, including imaging examinations and/or genetic testing, were performed for 22 of the 30 recurrent cases (73%). The genetic

https://mc.manuscriptcentral.com/bmjpo

Confidential: For Review Only

and α1-antitrypsin deficiency cases were identified by additional investigations after developing further episode(s). Apart from organic acidaemia cases, all cases with ≥2 recurrent episodes underwent one or more further investigation to exclude possible underlying aetiologies. Genetic screening for SPINK1 (Serine Peptidase Inhibitor, Kazal Type 1), PRSS1 (cationic trypsinogen gene) and CFTR (cystic fibrosis transmembrane conductance regulator) gene mutations was performed for 12 of 30 cases with recurrent AP (40%). Of those who had genetic testing, five tests were positive (42%): three SPINK1 and two PRSS1 mutations. Additional imaging identified one case of pancreas divisum. The gallstone-associated case was diagnosed at the first episode, the patient developing a further episode whilst awaiting surgery. Three children of the 30 with recurrence (10%) showed imaging and clinical signs of chronic pancreatitis.

Major complications

Acutely or within one year, 21/90 children (23%) developed one or more major complication (Table 4). Some children developed more than one: one with five complications, four having four, one had three and five had two. Ten children developed one major complication. If recurrence were added as a significant AP complication, the number developing complications would increase to 45 of 90 children (50%).

The most common significant complication reported at initial admission was pancreatic necrosis in eight (9%). The aetiological associations of AP in pancreatic necrosis cases were idiopathic (n=3), medication (two asparaginase, one sodium valproate-associated), gallstones (n=1) and viral (n=1). Three of these eight cases required intensive care therapy.

Confidential: For Review Only

All cases, except one, were treated conservatively. One child died within 10 hours of admission to paediatric intensive care unit without surgical intervention.

Other major complications included respiratory failure in seven (7%), hyperglycaemia which required insulin therapy at some stage in six (6%), circulatory collapse in four (4%), renal failure in two (2%), and gastrointestinal bleeding in two (2%).

The main reported medium-term complications were pseudocyst formation (n=9, 10%), diabetes requiring insulin therapy at one year (n=4, 4%) and maldigestion (n=1, 1%). The aetiological associations of AP in the nine pseudocyst cases were drug therapy (n=4) and one each for trauma, gallstones, propionic acidaemia, viral infection and idiopathic. Six of nine pseudocysts spontaneously resolved during follow-up; two had incomplete follow-up data and one required surgical intervention. One of the diabetes cases had no reported hyperglycaemia initially. The case of maldigestion was diagnosed by both abnormal stool elastase and failure to thrive.

Additional reported acute complications were hypocalcaemia (n=23, 24%), hypoalbuminemia (n=49, 52%), moderate amounts of free fluid collection in the abdomen (n=28, 30%), pleural effusion (n=13, 14%), lung consolidation (n=3, 3%), lung collapse (one case), deterioration of pre-existing renal dysfunction (n=2, 2%), superior mesenteric vein thrombosis (n=1, 1%) and decompensation of propionic acidaemia (n=1, 1%). Increased seizure activity developed in a child taken off sodium valproate. An episode of AP altered future planned chemotherapy treatment for all children with leukaemia.

Confidential: For Review Only

Discussion

This study demonstrates that AP associates with significant morbidity in the short and longer term carrying a small but significant mortality risk. Other than recurrence, over a fifth of patients develop one or more significant complications. This recapitulates the 20–25% reported in other previous paediatric studies.[4,11,12] As in several previous studies, pseudocyst formation was the most common major complication, identified in 10% in this current study.[12-14]

Pancreatic necrosis, organ failure and hyperglycaemia requiring insulin treatment are the most frequent significant complications at first admission. In the current study, pancreatic necrosis was reported in 9% of cases which was more than the 4.6% reported by Sepúlved and Guerrero-Lozano.[15] The current study also shows that pancreatic necrosis may occur with variable aetiological associations in agreement with Raizner et al.[16] Transient hyperglycaemia is quite frequent in children with AP, with six requiring insulin therapy in hospital. Four children (4%) developed diabetes requiring regular insulin treatment at one year. This figure is similar to the 4.5% reported by a retrospective study from the United States but less than the 8% reported in a previous UK study.[7,17]

Within the follow-up period, one child with methylmalonic acidaemia developed maldigestion and failure to thrive following a first mild episode of AP. However, reporting of only one patient with pancreatic exocrine insufficiency (PEI) is likely to be a significant underestimate, as several patients had endocrine insufficiency at diagnosis and one year later. In adults, it has been found that PEI occurs frequently in individuals recovering from severe AP.[18,19] Studies suggest an absence of clinical symptoms does not allow the

https://mc.manuscriptcentral.com/bmjpo

Confidential: For Review Only

prediction of a normal nutritional status or sufficient treatment in chronic pancreatitis patients.[20,21] Therefore, routine evaluation of exocrine pancreatic function using faecal elastase-1 is recommended after all cases of AP to detect any PEI and provide guidance in decisions on enzyme replacement therapy.[22-25]

This study also shows that hypocalcaemia is common acutely, particularly in severe cases, consistent with other reports.[11] No cases required treatment. Other common abnormalities not requiring specific interventions were hypoalbuminaemia (52%), abdominal free fluid collections (30%) and pleural effusions (14%). Future research may benefit from focusing on these common abnormalities and particularly their roles in the prediction of AP severity.

Recurrent pancreatitis is a problem following the first diagnosis of AP. One third of children developed recurrent episode(s) within one year, which is comparable to the 28% reported in Italy, 33% in Canada and 36% in Mexico.[26-28] However, it is higher than the 10% previously reported in Scotland, likely due to the significant change in the aetiological associations of AP in childhood, and also higher than the 17% reported from the United States.[2,29,8]

Idiopathic (43%), hereditary factors (17%), medication (17%) and organic acidaemias (13%) are the commonest associations of acute recurrent pancreatitis, similar to findings in other studies.[30-34] Some of the idiopathic cases may represent under-investigation for genetic or autoimmune causation. This study found relapse may follow both mild and moderate/severe initial AP episodes. However, the majority (83%) of patients who developed recurrence had mild first episodes. When combining both complications and recurrence, 50% (45/90) of the patients in this study developed either major complication(s)

Confidential: For Review Only

from the first episode and/or recurrent pancreatitis within a year from diagnosis. The high recurrence rate with AP observed in this study and several previous paediatric series suggests AP is not a benign disease, even if classified initially as mild.[8,31,33,35]

Two patients died which indicates that AP has a case fatality of ~2.0% in the UK. This is less than the 5.3% and 6.3% reported in two earlier UK series.[17,36] Case fatality figures correspond to the 2% reported by a Scottish report being in line with a review suggesting mortality is less than 5% in most cohorts.[6,29] It is noticeably lower than the 10% reported in adults.[37]

A study limitation was the possibility that diabetes in some cases was the result of a contemporaneous onset of autoimmune type 1 diabetes but data on antibody status was not collected in the survey. Reporting of this follow up paper was delayed due to the lead researcher being caught up in civil conflict, but we believe the data interpretation is still robust. Patients were reported by paediatricians or paediatric surgeons who are members of BAPS, we believe that the vast majority of cases in those <15 years who have been managed by these professionals. However, there may be a small proportion of patients who are managed solely by adult surgeons (or paediatric surgeons who are not members of BAPS) and would therefore be missed from reporting.

In summary, the present study suggests that AP is associated with significant medium-term morbidity. One year after diagnosis, only 56% of children have made a full recovery without residual problems; 44% of patients experience recurrence and/or other complications. Residual co-morbidities were identified in about one tenth of children. Even if classified initially as a mild episode, the prognosis for AP should be guarded.

Confidential: For Review Only

"What is already known on this topic"

- The clinical course and complications of AP in children are variable, ranging from mild self-limiting illness to organ failure and death.
- Following an episode of AP, children may develop further episodes.
- There has been no prospective study focusing on the complications and morbidities following AP in children and the incidence of these complications.

"What this study adds"

- The most common complications of AP were pancreatic necrosis initially and in the medium-term, pseudocyst formation and diabetes requiring insulin.
- Following AP, a significant minority of children will not make a full recovery without residual problems.
- Even if classified as mild, childhood AP can lead to significant complications and morbidities and should not be considered a benign condition in childhood.

Author contributions: A Bhanot wrote the preliminary draft paper under supervision from TC, AA Majbar conducted the BPSU study and performed the data analyses, JPHS developed the mechanics of the study, LP Hunt supported and contributed to the statistical analysis, EC and PJ edited the questionnaires and took part in diagnostic meeting sessions. All authors contributed to the final editing.

Acknowledgements: We need to acknowledge the significant contribution of Mr Richard Lynn, late of the RCPCH BPSU to the successful running of this study.

Confidential: For Review Only

This is an independent opinion from Bristol Biomedical Research Centre in the National Institute for Health Research Biomedical Research Centre and Unit Funding Scheme. The views expressed in this publication are those of the authors and not necessarily those of the NHS, the National Institute for Health Research or the Department of Health and Social Care.

Funding statement: This study was funded by the Libyan Ministry of Higher Education and Scientific Research represented by the Libyan Embassy in London as an MD scholarship for Dr A. Majbar at the University of Bristol. Additional funding came from the National Institute for Health Research Biomedical Research Centre Funding Scheme.

Confidential: For Review Only

References

1. Bradley EL. A clinically based classification system for acute pancreatitis. Summary of the International Symposium on Acute Pancreatitis, Atlanta, Ga, September 11 through 13, 1992. *The Archives of Surgery.* 1993; 128(5):586-90.
2. Majbar AA et al. Incidence and Clinical Associations of Childhood Acute Pancreatitis. *Pediatrics*. 2016; 138(3): :e20161198.
3. Chang YJ et al. Acute pancreatitis in children. *Acta Paediatrica*. 2011; 100(5):740-4.
4. Berney T et al. Influence of severe underlying pathology and hypovolemic shock on the development of acute pancreatitis in children. *Journal of Pediatric Surgery*. 1996; 31(9):1256-61.
5. Bai HX, Lowe ME and Husain SZ. What have we learned about acute pancreatitis in children? *Journal of Pediatric Gastroenterology and Nutrition*. 2011; 52(3):262-70.
6. Abu-El-Haija M et al. Management of Acute Pancreatitis in the Pediatric Population: A Clinical Report From the North American Society for Pediatric Gastroenterology, Hepatology and Nutrition Pancreas Committee. *Journal of Pediatric Gastroenterology and Nutrition.* 2018; 66(1):59–176.
7. Raman VS et al. Hyperglycemia and diabetes mellitus in children with pancreatitis. *The Journal of Pediatrics*. 2011; 158(4):612-616e1.
8. Sweeny KF et al. Rapid Progression of Acute Pancreatitis to Acute Recurrent Pancreatitis in Children. *Journal of Pediatric Gastroenterology and Nutrition.* 2019; 68(1):104-109.
9. Banks PA et al. Classification of acute pancreatitis--2012: revision of the Atlanta classification and definitions by international consensus. *Gut*. 2013; 62(1):102-11.
10. Morinville VD et al. Definitions of pediatric pancreatitis and survey of present clinical practices. *Journal of Pediatric Gastroenterology and Nutrition*. 2012; 55(3):261-5.
11. DeBanto JR et al. Acute pancreatitis in children. *The American Journal of Gastroenterology*. 2002; 97(7):1726-31.
12. Chang YJ et al. Acute pancreatitis in children. *Acta Paediatrica.* 2011; 100(5):740-4.
13. Das S et al. Pancreatic diseases in children in a north Indian referral hospital. *Indian Pediatrics*. 2004; 41(7):704-11.
14. Benifla M and Weizman Z, Acute pancreatitis in childhood: analysis of literature data. *Journal of Clinical Gastroenterology*. 2003; 37(2):169-72.
15. Sepúlved EVF and Guerrero-Lozano R. Acute pancreatitis and recurrent acute pancreatitis: an exploration of clinical and etiologic factors and outcomes. *Jornal de Pediatria.* 2019; 95(6):713-719.
16. Raizner A et al. Acute necrotizing pancreatitis in children. *The Journal of Pediatrics*. 2013; 162(4):788-92.
17. Sibert JR. Pancreatitis in childhood. *Postgraduate Medical Journal*. 1979; 55(641):171-5.

Confidential: For Review Only

18. Boreham B and Ammori BJ. A prospective evaluation of pancreatic exocrine function in patients with acute pancreatitis: correlation with extent of necrosis and pancreatic endocrine insufficiency. *Pancreatology*. 2003; 3(4):303-8.
19. Das SL et al. Relationship between the exocrine and endocrine pancreas after acute pancreatitis. *World Journal of Gastroenterology*. 2014; 20(45):17196-205.
20. Lindkvist B et al. Serum nutritional markers for prediction of pancreatic exocrine insufficiency in chronic pancreatitis. *Pancreatology.* 2012; 12(4):305-10.
21. Dominguez-Munoz JE and Iglesias-Garcia J. Oral pancreatic enzyme substitution therapy in chronic pancreatitis: is clinical response an appropriate marker for evaluation of therapeutic efficacy? *Jop*. 2010; 11(2):158-62.
22. Symersky T, van Hoorn B and Masclee AA. The outcome of a long-term follow-up of pancreatic function after recovery from acute pancreatitis. *Jop*. 2006; 7(5):447-53.
23. Pezzilli R et al. Exocrine pancreatic function during the early recovery phase of acute pancreatitis. *Hepatobiliary & Pancreatic Diseases International*. 2009; 8(3):316-9.
24. Vujasinovic M et al. Pancreatic exocrine insufficiency, diabetes mellitus and serum nutritional markers after acute pancreatitis. World Journal of Gastroenterology. 2014; 20(48):18432-8.
25. Loser C, Mollgaard A and Folsch UR. Faecal elastase 1: a novel, highly sensitive, and specific tubeless pancreatic function test. *Gut*. 1996; 39(4):580-6.
26. 26. Pezzilli R et al.Acute pancreatitis in children. An Italian multicentre study. *Digestive and Liver Disease*. 2002; 34(5):343-8.
27. Weizman Z and Durie PR. Acute pancreatitis in childhood. *The Journal of Pediatrics*. 1988; 113:24-9.
28. Sanchez-Ramirez CA et al. Acute and recurrent pancreatitis in children: etiological factors. *Acta Paediatrica*. 2007; 96(4):534-7.
29. Haddock G et al. Acute pancreatitis in children: a 15-year review. *Journal of Pediatric Surgery*. 1994; 29(6):719-22.
30. Randall et al. Pancreatitis in pre-adolescent children: a 10 year experience in the pediatric emergency department. *BMC Emergency Medicine*, 2019; 19:71.
31. Weizman Z and Durie PR. Acute pancreatitis in childhood. *The Journal of Pediatrics*. 1988; 113:24-9.
32. Werlin SL, Kugathasan S and Frautschy BC. Pancreatitis in children. *Journal of Pediatric Gastroenterology and Nutrition*. 2003; 37(5):591-5.
33. Sanchez-Ramirez CA et al. Acute and recurrent pancreatitis in children: etiological factors. *Acta Paediatrica*. 2007; 96(4):534-7.
34. Lucidi V et al. The etiology of acute recurrent pancreatitis in children: a challenge for pediatricians. *Pancreas*. 2011. 40(4):517-21.
35. Choi BH et al. Acute pancreatitis associated with biliary disease in children. *Journal of Gastroenterology and Hepatology*. 2003; 18(8):915-21.
36. Moossa AR. Acute pancreatitis in childhood. A study of 16 cases and review of the literature. Progress in Pediatric Surgery. 1972; 4:111-27.

Confidential: For Review Only

37. Williams JG et al. Gastroenterology services in the UK. The burden of disease, and the organisation and delivery of services for gastrointestinal and liver disorders: a review of the evidence. *Gut.* 2007; 56:1-113.

Confidential: For Review Only

Figures

Figure 1: Recruitment and inclusion process for new acute pancreatitis cases

Tables

Main association	Severity	
	**Moderate/severe (n=16)**<br>**No of cases (%)**	**Mild (n=78)**<br>**No of cases (%)**
**Idiopathic**	4 (25)	31 (40)
**Drugs**	6 (38)	12 (15)
**Gallstones**	1 (6)	11 (14)
**Hereditary**	1 (6)	6 (8)
**Organic acidaemia**	2 (13)	5 (6)
**Anatomical anomalies**	.	5 (6)
**Viral infections**	1 (6)	2 (3)
**Systemic diseases**	.	2 (3)
**Trauma**	1 (6)	.
**Others**	.	4 (5)

Table 1: Main associations of acute pancreatitis in severe and mild cases

Confidential: For Review Only

Outcome	No of cases (%)
Full recovery	53 (59.0)
Full recovery from the initial episode but with recurrence of pancreatitis	26 (29)
Residual problems without recurrence	5 (6)
Residual problems with recurrence	4 (4)

Table 2: Outcomes of 90 cases reported at one-year following acute pancreatitis

Number of episodes	No of children (%)
2	17 (57)
3	4 (13)
4	4 (13)
5	4 (13)
6	1 (3)

Table 3: Number of episodes of acute pancreatitis in the 30 patients who had a recurrence

Complication	No of cases* (%)
**Short term (at initial admission, n=94)**	
Pancreatic necrosis	8 (9)
Respiratory failure	7 (7%)
Hyperglycaemia required insulin therapy	6 (6%)
Circulatory collapse	4 (4%)
Renal failure	2 (2%)
Gastrointestinal bleeding	2 (2%)
**Medium and long term (n=90)**	
Pseudocyst formation	9 (10%)
Diabetes	4 (4%)
Maldigestion	1 (1%)

*some patients had more than one complication

Table 4: Major reported complications of acute pancreatitis

Confidential: For Review Only

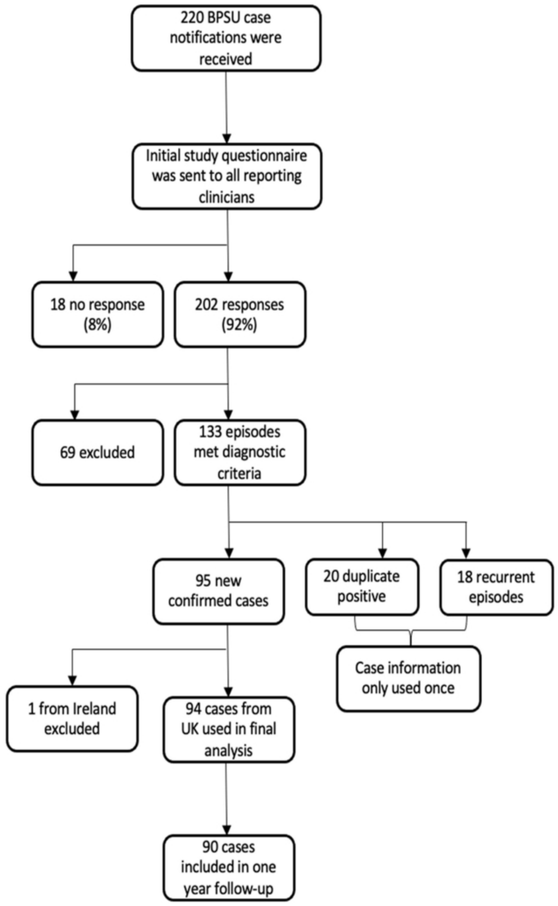

209x297mm (300 x 300 DPI)

BMJ Paediatrics Open

Long term morbidity and outcomes of acute pancreatitis in children

Journal:	*BMJ Paediatrics Open*
Manuscript ID	bmjpo-2022-001487.R1
Article Type:	Original research
Date Submitted by the Author:	06-Jun-2022
Complete List of Authors:	Bhanot, A; University of Bristol, Faculty of Health Sciences,<br>Majbar, AA; Sabratha Teaching Hospital, Department of Paediatrics<br>Candler, Toby; University Hospitals Bristol NHS Foundation Trust, Paediatric Diabetes and Endocrinology<br>Hunt, LP; University of Bristol, NIHR Bristol Biomedical Research Centre, Nutrition Theme<br>Cusick, E; University Hospitals Bristol NHS Foundation Trust<br>Johnson, Paul; University of Oxford, Nuffield Department of Surgery; NIHR Oxford Biomedical Research Centre<br>Shield, Julian; University of Bristol, Biomedical Research Unit in Nutrition; University Hospitals Bristol NHS Foundation Trust, Paediatric Diabetes and Endocrinology
Keywords:	Endocrinology, Gastroenterology

SCHOLARONE™
Manuscripts

I, the Submitting Author has the right to grant and does grant on behalf of all authors of the Work (as defined in the below author licence), an exclusive licence and/or a non-exclusive licence for contributions from authors who are: i) UK Crown employees; ii) where BMJ has agreed a CC-BY licence shall apply, and/or iii) in accordance with the terms applicable for US Federal Government officers or employees acting as part of their official duties; on a worldwide, perpetual, irrevocable, royalty-free basis to BMJ Publishing Group Ltd ("BMJ") its licensees and where the relevant Journal is co-owned by BMJ to the co-owners of the Journal, to publish the Work in this journal and any other BMJ products and to exploit all rights, as set out in our licence.

The Submitting Author accepts and understands that any supply made under these terms is made by BMJ to the Submitting Author unless you are acting as an employee on behalf of your employer or a postgraduate student of an affiliated institution which is paying any applicable article publishing charge ("APC") for Open Access articles. Where the Submitting Author wishes to make the Work available on an Open Access basis (and intends to pay the relevant APC), the terms of reuse of such Open Access shall be governed by a Creative Commons licence – details of these licences and which Creative Commons licence will apply to this Work are set out in our licence referred to above.

Other than as permitted in any relevant BMJ Author's Self Archiving Policies, I confirm this Work has not been accepted for publication elsewhere, is not being considered for publication elsewhere and does not duplicate material already published. I confirm all authors consent to publication of this Work and authorise the granting of this licence.

Confidential: For Review Only

One year follow up of children diagnosed with acute pancreatitis: co-morbidity and outcomes

Journal: BMJ Open Paediatrics

Title (max. 10 words)**: Acute pancreatitis in children: morbidity and outcomes at one year**

Authors: Bhanot A[*1], Majbar AA[*2], Candler TP[3], Hunt LP[4], Johnson PRV[56], Cusick E[3], Hamilton-Shield J[3,4].
*** Joint first Authors**
Word count: 2691 words
Abstract: 247 words
Tables/illustrations: 5
References: 37

1. Faculty of Health Sciences, University of Bristol, BS2 8DZ, UK
2. Department of Paediatrics, Sabratha Teaching Hospital, Sabratha, Libya
3. Bristol Royal Hospital for Children, Upper Maudlin Street, Bristol, BS28BJ
4. NIHR Bristol Biomedical Research Centre, Nutrition Theme, University of Bristol, BS1 2NT, UK
5. Academic Paediatric Surgery Unit, Nuffield Department of Surgical Sciences, University of Oxford, John Radcliffe Hospital, Oxford
6. NIHR Oxford Biomedical Research Centre

Correspondence to: Professor Julian Hamilton-Shield, Bristol Royal Hospital for Children, Upper Maudlin Street, Bristol, BS2 8BJ. or e-mail: j.p.h.shield@bristol.ac.uk

Confidential: For Review Only

Abstract

Objective:

To establish short- and medium-term complications one year post diagnosis, of acute pancreatitis (AP) in children aged 0 to 14 years.

Design:

One year follow-up of a prospective monthly surveillance of new cases of AP in children under 15 years through the British Paediatric Surveillance Unit (BPSU) from April 2013 to April 2014.

Setting:

A monthly surveillance of >3700 consultant paediatricians and paediatric surgeons in the United Kingdom and Ireland using the BPSU.

Patients:

Children aged 0-14 years with a new diagnosis of AP.

Main outcome measures:

The outcomes following AP, including the incidence of complications and co-morbidity at diagnosis and at one year.

Results:

Of the 94 new confirmed cases of AP identified in the UK during the study period, 90 cases (96%) were included in the one-year follow-up. 30 patients (32%) developed further episode(s) of AP. Over a fifth of patients developed one or more major complication. At initial admission, the most common of these was pancreatic necrosis (n=8, 9%), followed by respiratory failure (n=7, 7%). Reported complications by one year were pseudocyst formation (n=9, 10%), diabetes requiring insulin therapy (n=4, 4%) and maldigestion (n=1, 1%). At one year post diagnosis, only 59% of children made a full recovery with no acute or

https://mc.manuscriptcentral.com/bmjpo

Confidential: For Review Only

chronic complications or recurrent episodes of AP. Two patients died, indicating a case fatality of ~2.0%.

Conclusions:

Acute pancreatitis in childhood is associated with significant short- and medium-term complications and co-morbidities including risk of recurrence in approximately a third of cases.

Confidential: For Review Only

Introduction

Acute pancreatitis (AP) is an inflammatory condition classically presenting with sudden onset upper abdominal pain.[1] Our previous prospective study found an incidence of AP in UK children under 15 years of 0.78 per 100,000.[2] It is reported that 25% of children develop major complications following an episode of AP.[3,4] The clinical course and complications of AP in children are variable, ranging from mild self-limiting illness to organ failure and death.[5,6]

Pancreatic necrosis, pseudocyst formation and abscesses are some of the complications following AP.[6] Diabetes Mellitus (DM) after AP has also been reported in children, with one retrospective study in the United States reporting DM in 4.5% of cases.[7] Until now in the UK, there has been no prospective study focusing on the complications and co-morbidity following AP in children.

Following an episode of AP, children may develop further episodes.[5] Recurrence has been reported in 15–35% of children with AP.[6] One prospective study in the United States found that 17% of patients developed acute recurrent pancreatitis following an episode of AP.[8] However, the rate of recurrence following AP has yet to be determined in a prospective UK study.

The aim of this study was to conduct a prospective follow-up to our previous national-based analysis of AP in childhood to characterise the outcomes of AP, including determining the prevalence of complications and co-morbidity in the short to medium term.

Confidential: For Review Only

Methods

As previously described, a prospective study was carried out using the British Paediatric Surveillance Unit (BPSU) reporting system of the Royal College of Paediatrics and Child Health (RCPCH) to identify any new diagnosis of AP in 0-14 year old children in the UK and Ireland.[2] From April 2013 to April 2014, AP in children under the age of 15 years was included on the orange card sent monthly to all consultant paediatricians who undertake clinical work in the UK or the Republic of Ireland. To identify children directly referred to surgical departments, the British Association of Paediatric Surgeons (BAPS) supported this study and paediatric surgeons received the orange card during the study period.

Diagnosis of AP required at least two of the three following features: (1) Acute onset of upper abdominal pain; (2) Serum amylase and/or lipase raised ≥3 times the upper limit of normal local range; and (3) Imaging findings characteristic of AP. These criteria for the diagnosis of AP were adapted according to revised Atlanta criteria by the "Acute Pancreatitis Classification Working Group" and a consensus of the International Study Group of Paediatric Pancreatitis: In Search for a Cure (INSPPIRE)[9,10]. Clinicians returned the card notifying the BPSU of any AP cases seen or "nothing to report". The orange card return compliance was 95.3% during the study.

Each reporting clinician was sent an initial questionnaire to collect additional information on the clinical presentation, diagnosis, and management, as well as acute complications and mortality. Upon the questionnaire's completion, the eligibility of the patient was determined and the physicians' diagnoses were reviewed by the study investigators (A.A.M, J.P.H.S, E.C).[2] If a child had more than one episode of AP during the study period, only the

Confidential: For Review Only

first episode was included as a new case and the information on following episode(s) was used as follow-up data.

A second questionnaire was sent to the reporting clinician one year after the initial presentation for diagnostic review, establish management patterns, and identify associated morbidity and outcome. The follow-up questionnaire had four sections. Section A collected information about weight and height. Section B collected data on medium term complications. Section B1 asked about any new surgical interventions, as well as development of pseudocyst, its diagnosis and treatment. Section B2 asked about the development of diabetes, and/or maldigestion and its diagnosis and treatment. Section C detailed further investigations undertaken after initial investigation and Section D asked about recurrence and outcome. The initial questionnaire also collected data on acute complications and mortality.

Statistics

Data were analysed using the Statistical Package for the Social Sciences (SPSS) (Version 21.0. Armonk, NY: IBM Corp), Stata vs 12.1 (StataCorp LP, Texas, 1985-2011) and Microsoft Excel (2013).

The proportions of patients who were considered to have mild or moderate/severe disease were determined. Factors associated with developing AP were compared between mild and moderate/severe groups using Fisher's Exact test.

The percentages of patients developing short and medium-term complications up to one year post diagnosis of the first episode of AP were determined. Criteria for considering

https://mc.manuscriptcentral.com/bmjpo

Confidential: For Review Only

major complications of the first episode were: (1) all complications required to classify an episode as moderate/severe pancreatitis (organ failure, pancreatic necrosis, and pseudocyst formation), (2) complications requiring treatment at the initial admission (hyperglycaemia) and (3) residual disorders requiring treatment at the end of the follow-up period (diabetes and maldigestion). The major complications were divided into short-term (identified early during initial admission) and medium term (identified in patients with prolonged admission or after discharge during follow-up). Minor complications were also reported.

The percentage of patients who developed further episode(s) of AP within one year from diagnosis were identified, as well as the average number of recurrent episodes and reported associations.

The prognosis and medium-term outcomes at one year of the first episode of AP in children were evaluated by dividing the cohort into five outcome groups: (1) children with full recovery, (2) children with full recovery from the initial episode, but with recurrence of pancreatitis, (3) children with residual problems without recurrence, (4) children with residual problems and recurrence, and (5) children who died while hospitalised.

Ethics

The study was approved by the National Research Ethics Service Committee South West, Central Bristol (REC reference 11/SW/0132) and was granted Section 251 of the National Information Governance Board for Health and Social Care permission by its Ethics and Confidentiality Committee under reference ECC 6-02(FT12)/2012.

Confidential: For Review Only

Results

From April 2013 to April 2014, there were 94 new confirmed cases of AP identified. At one year follow-up, clinical data were available for 90 of these cases (96%), no responses were received for four cases.

Demographics

Female to male ratio was equal with 48/94 (51%) male. The median age at diagnosis was 11.2 years (IQR 7.1-14.4), range 1.3 to 14.9 years). White children accounted for 57 of 94 cases (61%), compared to 26 (28%) from Asian and five (5%) from African ethnic groups. Children of Pakistani origin accounted for the majority (18 of 26; 69%) of the patients of Asian heritage and 19% of the total cohort.[2]

Outcomes

Sixteen (17%) of the 94 new cases were considered to have moderate/severe disease. There was no association found between aetiology of AP and disease severity (P=0.22, Table 1). Two children (2.1%) died in hospital during their first admission: both had underlying chronic conditions (propionic acidaemia and cerebral palsy, global developmental delay, epilepsy and microcephaly). All children required hospital management; eight requiring intensive care. Eighty-six patients (91%) were treated conservatively during their initial hospitalisation. Twenty (21%) required one or more related surgical intervention during the initial admission and/or the follow-up period. The most common indications for surgical intervention were: diagnostic laparoscopies in seemingly idiopathic cases, elimination of the underlying predisposing factors such as gallstones and choledochal cysts, and treatment of complications (Table 2).

https://mc.manuscriptcentral.com/bmjpo

Confidential: For Review Only

The outcomes of the 90 cases included at one year are shown in Table 3. Residual co-morbidities included insulin-dependent diabetes (n=4), clinical and imaging features of chronic pancreatitis (n=3), maldigestion with failure to thrive (n=1) and residual radiological changes in the pancreas without significant clinical findings (n=1).

Recurrent disease

Thirty patients (33%) developed further episode(s) of AP within one year. These 30 had 88 episodes during the study period with an average of 2.9 episodes/patient (Table 4). Twenty-five of these (83%) had mild initial episodes.

The reported aetiology of AP in the recurrent group were idiopathic (n=13, 43%), genetic (n=5, 17%), drugs (n=5, 17%), organic acidaemias (n=4, 13%), pancreas divisum (n=1, 3%), gallstones (n=1, 3%) and α1-antitrypsin deficiency (n=1, 3%). Each of the five drug-associated cases (two asparaginase, one mercaptopurine, one carbamazepine and one opiates) developed one further episode. The recurrent episode in the child whose first episode was due to opiates was again attributed to opiates. The new episode in the child whose first episode was mercaptopurine-associated was ascribed to prednisolone treatment for acute lymphoblastic leukaemia. For the other three cases, available data did not confirm or discount whether the new episodes developed by reintroducing the same medication.

At least one imaging examination was performed during the first episode in the majority of this recurrent group (28 of 30; 93%). Further investigations, including imaging examinations

Confidential: For Review Only

and/or genetic testing, were performed for 22 of the 30 recurrent cases (73%). The genetic and α1-antitrypsin deficiency cases were identified by additional investigations after developing further episode(s). Apart from organic acidaemia cases, all cases with ≥2 recurrent episodes underwent one or more further investigation to exclude possible underlying aetiologies. Genetic screening for SPINK1 (Serine Peptidase Inhibitor, Kazal Type 1), PRSS1 (cationic trypsinogen gene) and CFTR (cystic fibrosis transmembrane conductance regulator) gene mutations was performed for 12 of 30 cases with recurrent AP (40%). Of those who had genetic testing, five tests were positive (42%): three SPINK1 and two PRSS1 mutations. Additional imaging identified one case of pancreas divisum. The gallstone-associated case was diagnosed at the first episode, the patient developing a further episode whilst awaiting surgery. Three children of the 30 with recurrence (10%) showed imaging and clinical signs of chronic pancreatitis.

Major complications

Acutely or within one year, 21/90 children (23%) developed one or more major complication (Table 5). Some children developed more than one: one with five complications, four having four, one had three and five had two. Ten children developed one major complication. If recurrence were added as a significant AP complication, the number developing complications would increase to 45 of 90 children (50%).

The most common significant complication reported at initial admission was pancreatic necrosis in eight (9%). The aetiological associations of AP in pancreatic necrosis cases were idiopathic (n=3), medication (two asparaginase, one sodium valproate-associated), gallstones (n=1) and viral (n=1). Three of these eight cases required intensive care therapy.

Confidential: For Review Only

All cases, except one, were treated conservatively. One child died within 10 hours of admission to paediatric intensive care unit without surgical intervention.

Other major complications included respiratory failure in seven (7%), hyperglycaemia which required insulin therapy at some stage in six (6%), circulatory collapse in four (4%), renal failure in two (2%), and gastrointestinal bleeding in two (2%).

The main reported medium-term complications were pseudocyst formation (n=9, 10%), diabetes requiring insulin therapy at one year (n=4, 4%) and maldigestion (n=1, 1%). The aetiological associations of AP in the nine pseudocyst cases were drug therapy (n=4) and one each for trauma, gallstones, propionic acidaemia, viral infection and idiopathic. Six of nine pseudocysts spontaneously resolved during follow-up; two had incomplete follow-up data and one required surgical intervention. One of the diabetes cases had no reported hyperglycaemia initially. The case of maldigestion was diagnosed by both abnormal stool elastase and failure to thrive.

Additional reported acute complications were hypocalcaemia (n=23, 24%), hypoalbuminemia (n=49, 52%), moderate amounts of free fluid collection in the abdomen (n=28, 30%), pleural effusion (n=13, 14%), lung consolidation (n=3, 3%), lung collapse (one case), deterioration of pre-existing renal dysfunction (n=2, 2%), superior mesenteric vein thrombosis (n=1, 1%) and decompensation of propionic acidaemia (n=1, 1%). Increased seizure activity developed in a child taken off sodium valproate. An episode of AP altered future planned chemotherapy treatment for all children with leukaemia.

Confidential: For Review Only

Discussion

This study demonstrates that AP associates with significant morbidity in the short and medium term carrying a small but significant mortality risk. Other than recurrence, over a fifth of patients develop one or more significant complications. This recapitulates the 20–25% reported in other previous paediatric studies.[4,11,12] As in several previous studies, pseudocyst formation was the most common major complication, identified in 10% in this current study, the majority of which resolved spontaneously within one year.[12-14]

Pancreatic necrosis, organ failure and hyperglycaemia requiring insulin treatment are the most frequent significant complications at first admission. In the current study, pancreatic necrosis was reported in 9% of cases which was more than the 4.6% reported by Sepúlved and Guerrero-Lozano.[15] The current study also shows that pancreatic necrosis may occur with variable aetiological associations in agreement with Raizner et al.[16] Transient hyperglycaemia is quite frequent in children with AP, with six requiring insulin therapy in hospital. Four children (4%) developed diabetes requiring regular insulin treatment at one year. This figure is similar to the 4.5% reported by a retrospective study from the United States but less than the 8% reported in a previous UK study.[7,17]

Within the follow-up period, one child with methylmalonic acidaemia developed maldigestion and failure to thrive following a first mild episode of AP. However, reporting of only one patient with pancreatic exocrine insufficiency (PEI) is likely to be a significant underestimate, as several patients had endocrine insufficiency at diagnosis and one year later. In adults, it has been found that PEI occurs frequently in individuals recovering from severe AP.[18,19] Studies suggest an absence of clinical symptoms does not allow the

Confidential: For Review Only

prediction of a normal nutritional status or sufficient treatment in chronic pancreatitis patients.[20,21] Therefore, routine evaluation of exocrine pancreatic function using faecal elastase-1 is recommended after all cases of AP to detect any PEI and provide guidance in decisions on enzyme replacement therapy.[22-25]

This study also shows that hypocalcaemia is common acutely, particularly in severe cases, consistent with other reports.[11] No cases required treatment. Other common abnormalities not requiring specific interventions were hypoalbuminaemia (52%), abdominal free fluid collections (30%) and pleural effusions (14%). Future research may benefit from focusing on these common abnormalities and particularly their roles in the prediction of AP severity.

Recurrent pancreatitis is a problem following the first diagnosis of AP. One third of children developed recurrent episode(s) within one year, which is comparable to the 28% reported in Italy, 33% in Canada and 36% in Mexico.[26-28] However, it is higher than the 10% previously reported in Scotland, likely due to the significant change in the aetiological associations of AP in childhood, and also higher than the 17% reported from the United States.[2,29,8]

Idiopathic (43%), hereditary factors (17%), medication (17%) and organic acidaemias (13%) are the commonest associations of acute recurrent pancreatitis, similar to findings in other studies.[30-34] Some of the idiopathic cases may represent under-investigation for genetic or autoimmune causation. This study found relapse may follow both mild and moderate/severe initial AP episodes. However, the majority (83%) of patients who developed recurrence had mild first episodes. When combining both complications and recurrence, 50% (45/90) of the patients in this study developed either major complication(s)

Confidential: For Review Only

from the first episode and/or recurrent pancreatitis within a year from diagnosis. The high recurrence rate with AP observed in this study and several previous paediatric series suggests AP is not a benign disease, even if classified initially as mild.[8,31,33,35]

Two patients died which indicates that AP has a case fatality of ~2.0% in the UK. This is less than the 5.3% and 6.3% reported in two earlier UK series.[17,36] Case fatality figures correspond to the 2% reported by a Scottish report being in line with a review suggesting mortality is less than 5% in most cohorts.[6,29] It is noticeably lower than the 10% reported in adults.[37]

A study limitation was the possibility that diabetes in some cases was the result of a contemporaneous onset of autoimmune type 1 diabetes but data on antibody status was not collected in the survey. Reporting of this follow up paper was delayed due to the lead researcher being caught up in civil conflict, but we believe the data interpretation is still robust. Patients were reported by paediatricians or paediatric surgeons who are members of BAPS. We believe that the vast majority of cases in those <15 years will have been managed by these professionals. However, there may be a small proportion of patients who are managed solely by adult surgeons (or paediatric surgeons who are not members of BAPS) and would therefore be missed from reporting. Furthermore, a degree of under-reporting is likely with any voluntary surveillance system.

In summary, the present study suggests that AP is associated with significant medium-term morbidity. One year after diagnosis, only 59% of children made a full recovery with no acute or chronic complications or recurrent episodes of AP. Recurrence and/or other

https://mc.manuscriptcentral.com/bmjpo

Confidential: For Review Only

complications were experienced by 41% of patients. Residual co-morbidities were identified in about one tenth of children. Even if classified initially as a mild episode, the prognosis for AP should be guarded.

"What is already known on this topic"

- The clinical course and complications of AP in children are variable, ranging from mild self-limiting illness to organ failure and death.
- Following an episode of AP, children may develop further episodes.
- There has been no prospective study focusing on the complications and morbidities following AP in children and the incidence of these complications.

"What this study adds"

- The most common complications of AP were pancreatic necrosis initially and in the medium-term, pseudocyst formation and diabetes requiring insulin.
- Following AP, a significant minority of children will not make a full recovery without residual problems.
- Even if classified as mild, childhood AP can lead to significant complications and morbidities and should not be considered a benign condition in childhood.

Author contributions: A Bhanot wrote the preliminary draft paper under supervision from TC, AA Majbar conducted the BPSU study and performed the data analyses, JPHS developed the mechanics of the study, LP Hunt supported and contributed to the statistical analysis, EC and PJ edited the questionnaires and took part in diagnostic meeting sessions. All authors contributed to the final editing.

https://mc.manuscriptcentral.com/bmjpo
Confidential: For Review Only

Acknowledgements: We need to acknowledge the significant contribution of Mr Richard Lynn, late of the RCPCH BPSU to the successful running of this study.

This is an independent opinion from Bristol Biomedical Research Centre in the National Institute for Health Research Biomedical Research Centre and Unit Funding Scheme. The views expressed in this publication are those of the authors and not necessarily those of the NHS, the National Institute for Health Research or the Department of Health and Social Care.

Funding statement: This study was funded by the Libyan Ministry of Higher Education and Scientific Research represented by the Libyan Embassy in London as an MD scholarship for Dr A. Majbar at the University of Bristol. Additional funding came from the National Institute for Health Research Biomedical Research Centre Funding Scheme.

Confidential: For Review Only

References

1. Bradley EL. A clinically based classification system for acute pancreatitis. Summary of the International Symposium on Acute Pancreatitis, Atlanta, Ga, September 11 through 13, 1992. *The Archives of Surgery.* 1993; 128(5):586-90.
2. Majbar AA et al. Incidence and Clinical Associations of Childhood Acute Pancreatitis. *Pediatrics*. 2016; 138(3): :e20161198.
3. Chang YJ et al. Acute pancreatitis in children. *Acta Paediatrica*. 2011; 100(5):740-4.
4. Berney T et al. Influence of severe underlying pathology and hypovolemic shock on the development of acute pancreatitis in children. *Journal of Pediatric Surgery*. 1996; 31(9):1256-61.
5. Bai HX, Lowe ME and Husain SZ. What have we learned about acute pancreatitis in children? *Journal of Pediatric Gastroenterology and Nutrition*. 2011; 52(3):262-70.
6. Abu-El-Haija M et al. Management of Acute Pancreatitis in the Pediatric Population: A Clinical Report From the North American Society for Pediatric Gastroenterology, Hepatology and Nutrition Pancreas Committee. *Journal of Pediatric Gastroenterology and Nutrition.* 2018; 66(1):59–176.
7. Raman VS et al. Hyperglycemia and diabetes mellitus in children with pancreatitis. *The Journal of Pediatrics*. 2011; 158(4):612-616e1.
8. Sweeny KF et al. Rapid Progression of Acute Pancreatitis to Acute Recurrent Pancreatitis in Children. *Journal of Pediatric Gastroenterology and Nutrition.* 2019; 68(1):104-109.
9. Banks PA et al. Classification of acute pancreatitis--2012: revision of the Atlanta classification and definitions by international consensus. *Gut*. 2013; 62(1):102-11.
10. Morinville VD et al. Definitions of pediatric pancreatitis and survey of present clinical practices. *Journal of Pediatric Gastroenterology and Nutrition*. 2012; 55(3):261-5.
11. DeBanto JR et al. Acute pancreatitis in children. *The American Journal of Gastroenterology*. 2002; 97(7):1726-31.
12. Chang YJ et al. Acute pancreatitis in children. *Acta Paediatrica.* 2011; 100(5):740-4.
13. Das S et al. Pancreatic diseases in children in a north Indian referral hospital. *Indian Pediatrics*. 2004; 41(7):704-11.
14. Benifla M and Weizman Z, Acute pancreatitis in childhood: analysis of literature data. *Journal of Clinical Gastroenterology*. 2003; 37(2):169-72.
15. Sepúlved EVF and Guerrero-Lozano R. Acute pancreatitis and recurrent acute pancreatitis: an exploration of clinical and etiologic factors and outcomes. *Jornal de Pediatria.* 2019; 95(6):713-719.
16. Raizner A et al. Acute necrotizing pancreatitis in children. *The Journal of Pediatrics*. 2013; 162(4):788-92.
17. Sibert JR. Pancreatitis in childhood. *Postgraduate Medical Journal*. 1979; 55(641):171-5.

Confidential: For Review Only

18. Boreham B and Ammori BJ. A prospective evaluation of pancreatic exocrine function in patients with acute pancreatitis: correlation with extent of necrosis and pancreatic endocrine insufficiency. *Pancreatology*. 2003; 3(4):303-8.
19. Das SL et al. Relationship between the exocrine and endocrine pancreas after acute pancreatitis. *World Journal of Gastroenterology*. 2014; 20(45):17196-205.
20. Lindkvist B et al. Serum nutritional markers for prediction of pancreatic exocrine insufficiency in chronic pancreatitis. *Pancreatology.* 2012; 12(4):305-10.
21. Dominguez-Munoz JE and Iglesias-Garcia J. Oral pancreatic enzyme substitution therapy in chronic pancreatitis: is clinical response an appropriate marker for evaluation of therapeutic efficacy? *Jop*. 2010; 11(2):158-62.
22. Symersky T, van Hoorn B and Masclee AA. The outcome of a long-term follow-up of pancreatic function after recovery from acute pancreatitis. *Jop*. 2006; 7(5):447-53.
23. Pezzilli R et al. Exocrine pancreatic function during the early recovery phase of acute pancreatitis. *Hepatobiliary & Pancreatic Diseases International*. 2009; 8(3):316-9.
24. Vujasinovic M et al. Pancreatic exocrine insufficiency, diabetes mellitus and serum nutritional markers after acute pancreatitis. World Journal of Gastroenterology. 2014; 20(48):18432-8.
25. Loser C, Mollgaard A and Folsch UR. Faecal elastase 1: a novel, highly sensitive, and specific tubeless pancreatic function test. *Gut*. 1996; 39(4):580-6.
26. 26. Pezzilli R et al.Acute pancreatitis in children. An Italian multicentre study. *Digestive and Liver Disease*. 2002; 34(5):343-8.
27. Weizman Z and Durie PR. Acute pancreatitis in childhood. *The Journal of Pediatrics*. 1988; 113:24-9.
28. Sanchez-Ramirez CA et al. Acute and recurrent pancreatitis in children: etiological factors. *Acta Paediatrica*. 2007; 96(4):534-7.
29. Haddock G et al. Acute pancreatitis in children: a 15-year review. *Journal of Pediatric Surgery*. 1994; 29(6):719-22.
30. Randall et al. Pancreatitis in pre-adolescent children: a 10 year experience in the pediatric emergency department. *BMC Emergency Medicine*, 2019; 19:71.
31. Weizman Z and Durie PR. Acute pancreatitis in childhood. *The Journal of Pediatrics*. 1988; 113:24-9.
32. Werlin SL, Kugathasan S and Frautschy BC. Pancreatitis in children. *Journal of Pediatric Gastroenterology and Nutrition*. 2003; 37(5):591-5.
33. Sanchez-Ramirez CA et al. Acute and recurrent pancreatitis in children: etiological factors. *Acta Paediatrica*. 2007; 96(4):534-7.
34. Lucidi V et al. The etiology of acute recurrent pancreatitis in children: a challenge for pediatricians. *Pancreas*. 2011. 40(4):517-21.
35. Choi BH et al. Acute pancreatitis associated with biliary disease in children. *Journal of Gastroenterology and Hepatology*. 2003; 18(8):915-21.
36. Moossa AR. Acute pancreatitis in childhood. A study of 16 cases and review of the literature. Progress in Pediatric Surgery. 1972; 4:111-27.

Confidential: For Review Only

37. Williams JG et al. Gastroenterology services in the UK. The burden of disease, and the organisation and delivery of services for gastrointestinal and liver disorders: a review of the evidence. *Gut.* 2007; 56:1-113.

Confidential: For Review Only

Figures

Figure 1: Recruitment and inclusion process for new acute pancreatitis cases

Tables

Main association	Severity	
	Moderate/severe (n=16)<br>No of cases (%)	Mild (n=78)<br>No of cases (%)
Idiopathic	4 (25)	31 (40)
Drugs	6 (38)	12 (15)
Gallstones	1 (6)	11 (14)
Hereditary	1 (6)	6 (8)
Organic acidaemia	2 (13)	5 (6)
Anatomical anomalies	.	5 (6)
Viral infections	1 (6)	2 (3)
Systemic diseases	.	2 (3)
Trauma	1 (6)	.
Others	.	4 (5)

Table 1: Main associations of acute pancreatitis in severe and mild cases

Confidential: For Review Only

No	Gender. Age (years)	Association	Intervention(s)
1	Male, 2.3	Methylmalonic acidaemia	1. ERCP sphincterotomy**
2	Female, 12.6	Gallstones	1. ERCP sphincterotomy and stones removal **<br>2. Elective cholecystectomy **
3	Male, 13	Gallstones	1. Elective cholecystectomy**
4	Female, 13.7	Pancreas divisum	1. ERCP, pancreatic duct drainage, sphincterotomy and stent insertion**
5	Male, 14.1	Sodium valproate	1. Percutaneous drainage of Left sided abdominal collection**
6	Male, 14.8	Gallstones	1. Laparoscopic cholecystectomy*
7	Female, 8	Choledochal cyst	1. Excision of type I fusiform choledochal malformation**
8	Male, 4.9	Idiopathic	1. ERCP, cyst-duodenostomy stent (Stent removed)**
9	Male, 10.3	Trauma	1. Endoscopic stent for pseudocyst* (unsuccessful)<br>2. Laparoscopic cystgastrotomy for pseudocyst*
10	Female, 13.5	Gallstones	1.Elective laparoscopic cholecystectomy**
11	Male, 2	Asparaginase	1. Diagnostic laparoscopy*
12	Female, 14.8	Gallstones	1. Elective cholecystectomy**
13	Female, 13.5	Gallstones	1. Cholecystectomy and Splenectomy**
14	Male, 14.6	Gallstones	1. ERCP sphincterotomy and stones removal*<br>2. Laparoscopic cholecystectomy**
15	Male, 5.5	Choledochal cyst	1. Excision of choledochal malformation with hepaticojejunostomy**
16	Female, 12	Viral infection	1. Laparotomy and drainage of large peripancreatic collection in lesser sac*
17	Male, 5.4	Hereditary	1. ERCP and stent placement*<br>2. ERCP and stent removal**
18	Female, 12	Idiopathic	1. Diagnostic laparoscopy for acute abdomen*
19	Male, 14.7	Gallstones	1. Elective laparoscopic cholecystectomy**
20	Male, 14.6	Gallstones	1. ERCP and removal of 6 CBD stones*

Table 2: Surgical and interventional procedures performed in 20 patients

*intervention at initial admission; **intervention during the follow-up period. Abbreviations: CBD, common bile duct; ERCP, endoscopic retrograde cholangiopancreatography.

Confidential: For Review Only

Outcome	No of cases (%)
Full recovery	53 (59)
Full recovery from the initial episode but with recurrence of pancreatitis	26 (29)
Residual problems without recurrence	5 (6)
Residual problems with recurrence	4 (4)
**Died in hospital**	2 (2)

Table 3: Outcomes of 90 cases reported at one-year following acute pancreatitis

Number of episodes	No of children (%)
2	17 (57)
3	4 (13)
4	4 (13)
5	4 (13)
6	1 (3)

Table 4: Number of episodes of acute pancreatitis in the 30 patients who had a recurrence

Complication	No of cases* (%)
**Short term (at initial admission, n=94)**	
Pancreatic necrosis	8 (9)
Respiratory failure	7 (7%)
Hyperglycaemia required insulin therapy	6 (6%)
Circulatory collapse	4 (4%)
Renal failure	2 (2%)
Gastrointestinal bleeding	2 (2%)
**Medium term (n=90)**	
Pseudocyst formation	9 (10%)
Diabetes	4 (4%)
Maldigestion	1 (1%)

*some patients had more than one complication

Table 5: Major reported complications of acute pancreatitis

Confidential: For Review Only

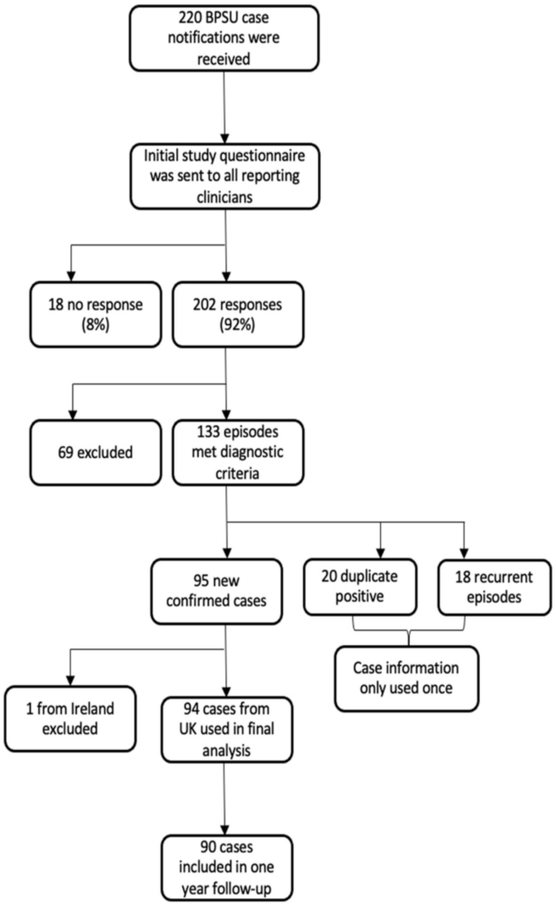

209x297mm (300 x 300 DPI)
